# Vitamin D and Its Analogues Decrease Amyloid-β (Aβ) Formation and Increase Aβ-Degradation

**DOI:** 10.3390/ijms18122764

**Published:** 2017-12-19

**Authors:** Marcus O. W. Grimm, Andrea Thiel, Anna A. Lauer, Jakob Winkler, Johannes Lehmann, Liesa Regner, Christopher Nelke, Daniel Janitschke, Céline Benoist, Olga Streidenberger, Hannah Stötzel, Kristina Endres, Christian Herr, Christoph Beisswenger, Heike S. Grimm, Robert Bals, Frank Lammert, Tobias Hartmann

**Affiliations:** 1 Experimental Neurology, Saarland University, Kirrberger Str. 1, 66421 Homburg/Saar, Germany; andrea.thiel@uks.eu (A.T.); Anna.Lauer@uks.eu (A.A.L.); jakob.winkler@uks.eu (J.W.); Johannes.Lehmann@uks.eu (J.L.); liesa.regner@uks.eu (L.R.); s8chnelk@stud.uni-saarland.de (C.N.); Daniel.janitschke@uks.eu (D.J.); s8cebeno@stud.uni-saarland.de (C.B.); olga.streidenberger@uks.eu (O.S.); hannah.stoetzel@uks.eu (H.S.); heike.grimm@uks.eu (H.S.G.); tobias.hartmann@uniklinikum-saarland.de (T.H.); 2 Neurodegeneration and Neurobiology, Saarland University, Kirrberger Str. 1, 66421 Homburg/Saar, Germany; 3 Deutsches Institut für DemenzPrävention (DIDP), Saarland University, Kirrberger Str. 1, 66421 Homburg/Saar, Germany; 4 Department of Internal Medicine II–Gastroenterology, Saarland University Hospital, Saarland University, Kirrberger Str. 100, 66421 Homburg/Saar, Germany; frank.lammert@uks.eu; 5 Department of Psychiatry and Psychotherapy, Clinical Research Group, University Medical Centre Johannes Gutenberg, University of Mainz, Untere Zahlbacher Str. 8, 55131 Mainz, Germany; kristina.endres@unimedizin-mainz.de; 6 Department of Internal Medicine V–Pulmonology, Allergology, Respiratory Intensive Care Medicine, Saarland University Hospital, Kirrberger Str. 1, 66421 Homburg/Saar, Germany; christian.herr@uks.eu (C.H.); christoph.beisswenger@uks.eu (C.B.); robert.bals@uks.eu (R.B.)

**Keywords:** vitamin D, vitamin D analogues, amyloid precursor protein, amyloid-β, secretases, Aβ-degradation

## Abstract

Alzheimer’s disease (AD) is characterized by extracellular plaques in the brain, mainly consisting of amyloid-β (Aβ), as derived from sequential cleavage of the amyloid precursor protein. Epidemiological studies suggest a tight link between hypovitaminosis of the secosteroid vitamin D and AD. Besides decreased vitamin D level in AD patients, an effect of vitamin D on Aβ-homeostasis is discussed. However, the exact underlying mechanisms remain to be elucidated and nothing is known about the potential effect of vitamin D analogues. Here we systematically investigate the effect of vitamin D and therapeutically used analogues (maxacalcitol, calcipotriol, alfacalcidol, paricalcitol, doxercalciferol) on AD-relevant mechanisms. D_2_ and D_3_ analogues decreased Aβ-production and increased Aβ-degradation in neuroblastoma cells or vitamin D deficient mouse brains. Effects were mediated by affecting the Aβ-producing enzymes BACE1 and γ-secretase. A reduced secretase activity was accompanied by a decreased BACE1 protein level and nicastrin expression, an essential component of the γ-secretase. Vitamin D and analogues decreased β-secretase activity, not only in mouse brains with mild vitamin D hypovitaminosis, but also in non-deficient mouse brains. Our results further strengthen the link between AD and vitamin D, suggesting that supplementation of vitamin D or vitamin D analogues might have beneficial effects in AD prevention.

## 1. Introduction

The most important pathological hallmarks of Alzheimer’s disease (AD), which is a progressive neurodegenerative disorder, are extracellular plaques that are composed of aggregated Aβ peptides and intracellular neurofibrillary tangles, consisting of hyperphosphorylated Tau proteins [1,2,3,4]. Aβ-generation depends on initial cleavage of the amyloid precursor protein (APP) by β-secretase 1 (BACE1), followed by intramembrane cleavage of APP by γ-secretase, a heterotetrameric protein complex consisting of presenilin 1 or 2 (PS1, PS2), nicastrin, anterior-pharynx-defective 1a or 1b (Aph1a, Aph1b), and presenilin-enhancer 2 (PEN2) [5,6,7,8]. Besides amyloidogenic, Aβ-releasing processing of APP, APP can be shed by α-secretases in a non-amyloidogenic pathway [9,10,11,12]. The α-secretases cleave APP within the Aβ domain and preclude the formation of Aβ peptides. Total Aβ level is not only dependent on the proteolytic activities of the APP cleaving secretases, but also on Aβ-degradation, involving e.g., the Aβ degrading enzymes neprilysin (NEP) and insulin-degrading enzyme (IDE) [13,14]. In addition to several lipids that influence the generation, degradation, and aggregation of Aβ peptides [15,16,17,18,19,20,21,22,23,24,25], it has recently been shown that fat soluble vitamins affect molecular mechanisms that are involved in AD pathogenesis, e.g., Aβ-induced neurotoxicity, oxidative stress, inflammatory processes, as well as Aβ-generation, Aβ-degradation, and Aβ-clearance [26,27,28,29,30,31]. Several vitamins, including vitamin A, provitamin Aβ-carotene, vitamin D_3_, vitamin K, and vitamin E, have also been reported to be reduced in plasma/serum of AD patients [30,32,33,34,35,36] and vitamin D hypovitaminosis affects up to 90% of the elderly population [37]. The biological activity of vitamin D can be attributed to binding interactions with the vitamin D receptor (VDR), which undergoes a conformational change, allowing for an interaction with the retinoid X receptor (RXR). The VDR-RXR heterodimer is considered to be the active complex that binds to vitamin D response elements in the DNA of target genes [38,39]. The VDR as well as the 1α-hydroxylase (CYP27B1), which converts 25(OH) vitamin D_3_ (calcifediol) into its active form 1,25(OH)_2_ vitamin D_3_ (calcitriol), have been shown to be expressed in human brain [40], and vitamin D and vitamin D metabolites have been reported to cross the blood-brain-barrier [41]. Recently, we and others could show that vitamin D deficiency causes an increase in amyloidogenic β-secretase cleavage of APP and a decrease in Aβ-degradation, resulting in elevated Aβ level [29,42]. In line, 25(OH) vitamin D supplementation elevated Aβ-degradation due to increased *NEP* expression and activity [29], supporting vitamin D supplementation as a novel approach to treat AD. In the present study, we compare these effects as mediated by vitamin D with vitamin D analogues on their amyloidogenic potential by investigating the APP processing pathways, as well as Aβ-degradation. Experiments were performed in neuroblastoma cells, revealing a concentration of approximately 2.5 ng/mL 25(OH) vitamin D_3_ [29]. However, it has to be pointed out that, although neuroblastoma cell lines have some neuronal properties, substantial differences when compared to neurons exist, which is a caveat of the study. Therefore, the main results were also validated ex vivo in homogenates of wildtype (wt) and hypovitaminosis D mouse brains. Further studies are needed to verify the potential positive effects of vitamin D and its analogues in vivo.

**Figure 1:**
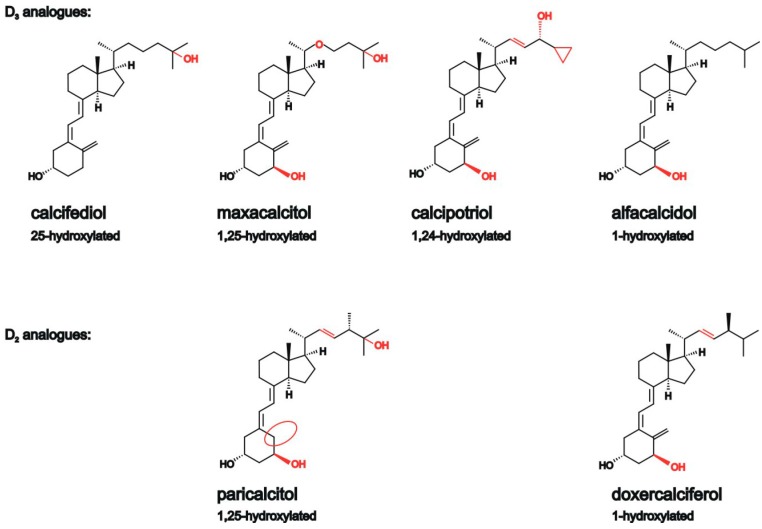
Chemical structure of 25(OH) vitamin D_3_ and different vitamin D analogues. Structural changes between the analogues are highlighted in red or with red cycles.

We selected 25(OH) vitamin D_3_ (calcifediol), which is converted by CYP27B1 to 1,25-dihydroxyvitamin D_3_, the natural vitamin D hormone, and therapeutically used analogues of vitamin D_3_ and D_2_ (Figure 1). Vitamin D analogues are modified in the side-chain portion of the molecule and exert a lower calcemic activity than natural vitamin D_3_, but retain many therapeutic properties of 1,25-dihydroxyvitamin D_3_. The 1,25-hydroxylated vitamin D_3_ analogue, maxacalcitol, is used to treat renal patients that are affected by secondary hyperparathyroidism, while the 1,24-hydroxylated vitamin D_3_ analogue calcipotriol for treatment of psoriasis and 1-hydroxylated alfacalcidol is administered to treat osteoporosis and secondary hyperparathyroidism. Paricalcitol and doxercalciferol were selected as vitamin D_2_ analogues. Identical to the vitamin D_3_ analogue maxacalcitol, paricalcitol contains a hydroxyl group at C_1_ and C_25_, but a vitamin D_2_ instead of a vitamin D_3_ side chain. 1-hydroxylated doxercalciferol is the vitamin D_2_ analogue that is comparable to the vitamin D_3_ analogue alfacalcidol in regard of the hydroxylation status. Both vitamin D_2_ analogues are used to treat elevated serum parathyroid hormone levels that are associated with secondary hyperparathyroidism [43,44].

## 2. Results

### 2.1. Vitamin D Analogues Decrease Total Aβ Level

In order to analyze whether analogues of vitamin D_3_ and vitamin D_2_ have similar anti-amyloidogenic properties when compared to 25(OH) vitamin D_3_ [29], we examined total secreted Aβ level in the human neuroblastoma cell line SH-SY5Y stably transfected with human APP695, the major isoform that is found in neurons [45], in the presence of calcifediol (25(OH) vitamin D_3_), maxacalcitol, calcipotriol, and alfacalcidol (vitamin D_3_ analogues), as well as the vitamin D_2_ analogues paricalcitol and doxercalciferol (Figure 1). We decided to use 25(OH) vitamin D_3_ instead of active 1,25(OH)_2_ vitamin D_3_ as calcifediol has a long serum half-life of approximately three weeks when compared to the short serum half-life (4–6 h) of 1,25(OH)_2_ vitamin D_3_ [46]. Cells were treated for 24 h in presence of vitamin D or analogues and secreted Aβ level were examined by analyzing the conditioned media of treated cells or control cells, incubated with the solvent EtOH. Vitamin D and its analogues were incubated in a final concentration of 100 nM. The concentration that was used corresponds to physiological serum concentrations of approximately 75 nmol/L [47,48], and is frequently used for cell culture experiments [26,49,50]. Furthermore, no significant change in cell viability was found using 100 nM vitamin D as compared to the solvent control (supplement Figure S1). Moreover, it has to be mentioned that calcifediol is used as a therapeutical supplement to treat vitamin D hypovitaminosis [51].

**Figure 2:**
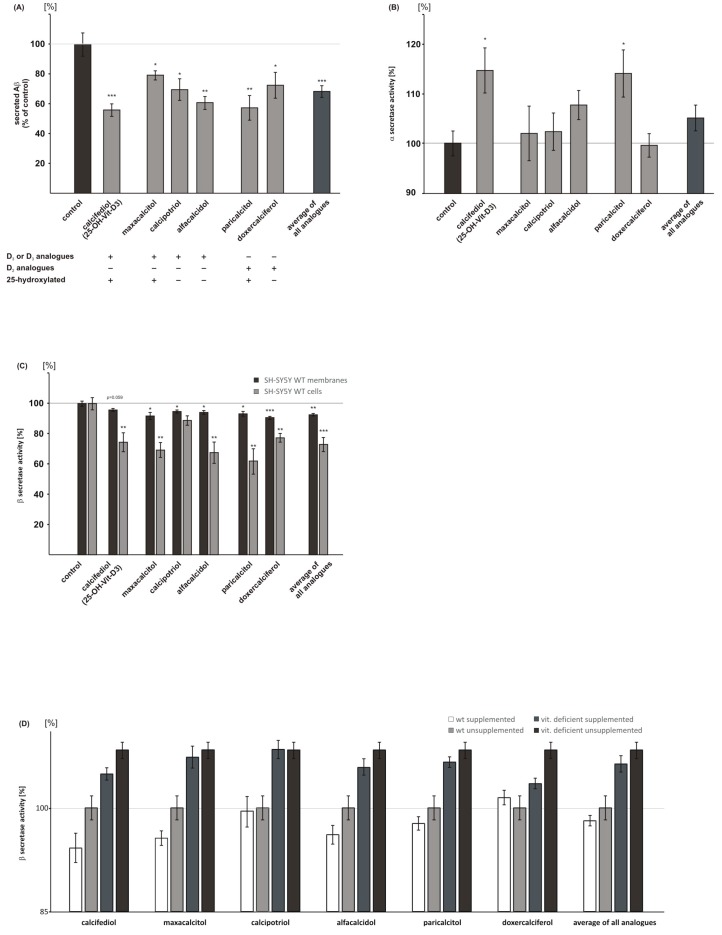

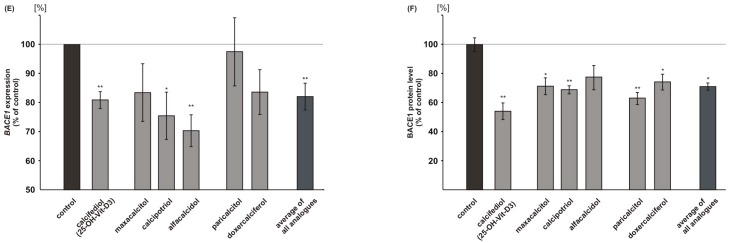
Effect of vitamin D and analogues on Aβ generation. Cells were treated with 25(OH) vitamin D_3_ (calcifediol), the vitamin D_3_ analogues maxacalcitol, calcipotriol, alfacalcidol, the vitamin D_2_ analogues paricalcitol, doxercalciferol in a final concentration of 100 nM or solvent control (EtOH). (**A**) Total secreted Aβ level in SH-SY5Y APP695 overexpressing cells (*n* = 3). Aβ of the conditioned media was analyzed by immunoprecipitation and Western Blot (WB) analysis. Using Post Hoc analysis, no significant differences between calcifediol and analogues were found in respect to their potential to reduce Aβ level. (**B**) Determination of α-secretase activity in living SH-SY5Y wt cells (*n* = 7). (**C**) Analysis of β-secretase activity in isolated membranes of SH-SY5Y wt cells (*n* = 7) and in living cells (*n* = 5). (**D**) β-secretase activity in three wt mouse brain and five vitamin D deficient mouse brain homogenates (*n* = 3). Vitamin D and analogues influence β-secretase activity in wt mouse brains and in vitamin D deficient mouse brains. (**E**) RT-PCR analysis of *BACE1* in SH-SY5Y wt cells (*n* = 3). (**F**) Determination of BACE1 protein level in cell lysates of SH-SY5Y wt cells by WB analysis (*n* = 3). Control conditions were set to 100% and illustrated as a line in the graphic. Error bars represent the standard error of the mean. Asteriks show the statistical significance calculated by unpaired Student’s *t* test (* *p* ≤ 0.05; ** *p* ≤ 0.01; *** *p* ≤ 0.001).

In presence of 25(OH) vitamin D_3_, calcifediol, which is converted by 1α-hydroxylase CYP27B1 to 1,25-dihydroxyvitamin D_3_, we found a significant reduction to 55.1% in total Aβ level when compared to cells that are treated with the solvent control (calcifediol: 55.1 ± 4.2%, *p* ≤ 0.001) (Figure 2A). The vitamin D_3_ analogues maxacalcitol (1,25-hydroxylated) and calcipotriol (1,24-hydroxylated) also showed significantly reduced Aβ level to 78.2% and 68.2%, respectively (maxacalcitol: 78.2 ± 3.1%, *p* = 0.031; calcipotriol: 68.2 ± 7.2%, *p* = 0.017). Alfacalcidol, a 1-hydroxylated vitamin D_3_ analogue, decreased total Aβ level to 60.3% (alfacalcidol: 60.3 ± 4.3%, *p* = 0.002). Vitamin D_2_ analogues also significantly reduced the Aβ level. The determination of total Aβ level of paricalcitol treated cells, a 1,25-hydroxylated vitamin D_2_ analogue, revealed a significant reduction to 56.3% (paricalcitol: 56.3 ± 8.2%, *p* = 0.005). The 1-hydroxylated vitamin D_2_ analogue doxercalciferol revealed a significant decrease to 70.9% (doxercalciferol: 70.9 ± 8.6%, *p* = 0.037). On average, mean analogues of vitamin D_3_ and D_2_ significantly reduced total Aβ level to 66.8% (mean analogues: 66.8 ± 3.9%, *p* ≤ 0.001) (Figure 2A). However, no significant differences in the Aβ level were obtained between single vitamin analogues as determined by ANOVA analysis.

### 2.2. Analysis of Non-Amyloidogenic APP Shedding in Presence of Vitamin D Analogues

Reduced total Aβ level can be caused by different mechanisms, including an increase in the non-amyloidogenic α-secretase dependent processing of APP. Therefore, we examined α-secretase activity in presence of 25(OH) vitamin D_3_ (calcifediol), vitamin D_3_ analogues, and vitamin D_2_ analogues in living SH-SY5Y wt cells. The vitamin D_3_ analogues maxacalcitol, calcipotriol, and alfacalcidol and the vitamin D_2_ analogue doxercalciferol showed no significant effect on α-secretase activity (Figure 2B). In contrast, 25(OH) vitamin D_3_ calcifediol and the vitamin D_2_ analogue paricalcitol, being also hydroxylated at C_25_ like calcifediol, revealed a significant increase in α-secretase activity, to 114.5% and 113.9%, respectively (calcifediol: 114.5 ± 4.5%, *p* = 0.015; paricalcitol: 113.9 ± 4.7%, *p* = 0.023). 

### 2.3. Vitamin D Analogues Decrease Amyloidogenic β-Secretase Dependent APP Cleavage

Cleavage of APP by BACE1 is the initial step in Aβ-production, generating the N-terminus of Aβ peptides. To evaluate whether vitamin D analogues have a direct effect on β-secretase activity, we prepared purified membranes of SH-SY5Y wt cells, incubated them with the vitamin D analogues, and measured β-secretase activity. 25(OH) vitamin D_3_, calcifediol, as well as all vitamin D analogues only slightly affected β-secretase activity directly, however, except for calcifediol, the observed reduction in β-secretase activity was statistically significant (maxacalcitol: 91.7 ± 2.3%, *p* = 0.016; calcipotriol: 94.7 ± 1.0%, *p* = 0.023; alfacalcidol: 94.1 ± 1.3%, *p* = 0.018; paricalcitol: 93.2 ± 1.6%, *p* = 0.013; and doxercalciferol: 90.7 ± 1.0%, *p* ≤ 0.001) (Figure 2C). To validate these results, we analyzed β-secretase activity in living SH-SY5Y wt cells. Vitamin D or vitamin D analogue supplemented cells revealed a more pronounced effect on β-secretase activity. Calcifediol significantly reduced β-secretase activity to 74.4%, the vitamin D_3_ analogues maxacalcitol and alfacalcidol showed a reduction to 69.2% and 67.5%, respectively (calcifediol: 74.4 ± 6.3%, *p* = 0.009; maxacalcitol: 69.2 ± 5.0%, *p* = 0.0014; alfacalcidol: 67.5 ± 7.1%, *p* = 0.004) (Figure 2C). Calcipotriol showed a non-significant decrease in β-secretase activity to 88.8% (calcipotriol: 88.8 ± 3.1%, *p* = 0.06). Additionally, the vitamin D_2_ analogues, paricalcitol and doxercalciferol, provided with a markedly reduction to 61.9% and 77.3%, a more pronounced effect in metabolically active cells when compared to β-secretase measurement of supplemented isolated membranes (Figure 2C). This indicates that beside the direct effect, indirect mechanisms, like gene expression or protein stability, contribute to the observed decrease in β-secretase activity. This observation is substantiated by the finding that all of the analogues averaged significantly reduced β-secretase activity in living cells to 72.9% (mean analogues, living cells: 72.9 ± 4.7%, *p* ≤ 0.001), whereas this effect was alleviated in direct β-secretase activity measurements (mean analogues, cellular membranes: 92.5 ± 0.8%, *p* = 0.004) (Figure 2C). Notably, calcifediol treated cells, which showed a reduction in β-secretase activity to 74.4% in living cells, reveal a nearly identical decrease in soluble β-secreted APP (sAPPβ) level to 76.9% (sAPPβ: 76.9 ± 1.8%, *p* = 0.0018) (supplement Figure S2), further underlining the data observed by the fluorescence resonance energy transfer (FRET)-based β-secretase assay. 

To further examine the potential of vitamin D analogues to decrease Aβ level by reducing β-secretase activity in an ex vivo situation, we analyzed vitamin D deficient mouse brains showing a 23% reduction in the 25(OH) vitamin D level as compared to wt mouse brains [29], either supplemented with vitamin D or unsupplemented. Therefore, we used homogenates of vitamin D deficient and wt mouse brains, and measured β-secretase activity in the presence or absence of vitamin D analogues. As already shown in our previous study [29] we found a significant increase in β-secretase activity in vitamin D deficient mouse brains when compared to wt mouse brains (Figure 2D). Supplementing of vitamin D deficient mouse brains with vitamin D_3_ or vitamin D_3_ and D_2_ analogues revealed the following results: five out of six analyzed vitamins showed a decrease in β-secretase activity, resulting in a partial rescue of β-secretase activity obtained for unsupplemented wt mouse brains. However, only calcifediol and doxercalciferol reached a level of significance (calcifediol, *p* = 0.026; doxercalciferol: *p* = 0.040; Table 1). Four out of six vitamins/analogues tended to decrease β-secretase activity when supplemented on wt mouse brains; three vitamin D species, calcifediol, alfacalcidol and maxacalcitol, revealed a significant reduction in β-secretase activity (calcifediol, *p* = 0.028; alfacalcidol, *p* = 0.050; maxacalcitol, *p* = 0.015). Analyzing the effect of all of the analogues averaged on either wt mouse brains or vitamin D deficient mouse brains, we found a similar decrease in β-secretase activity (Figure 2D), suggesting that both individuals with hypovitaminosis and individuals with normal vitamin D status, might profit from vitamin D supplementation in a similar range, which should be evaluated in further studies. A more detailed statistical analysis is given in Table 1 and Table 2.

As described above, we observed a more pronounced reduction in β-secretase activity in living cells in presence of vitamin D analogues when compared to the direct effect of these substances on β-secretase activity. To examine whether vitamin D analogues decrease β-secretase activity by affecting the gene expression of *BACE1*, we performed qPCR analysis of *BACE1* and determined BACE1 protein level by WB analysis in SH-SY5Y wt cells. Except for paricalcitol, all vitamin D_3_ and vitamin D_2_ analogues, as well as calcifediol, tended to decrease *BACE1* gene expression (Figure 2E). 

Significant results were only obtained for calcifediol, calcipotriol, and alfacalcidol (calcifediol: 80.8 ± 2.9%, *p* = 0.003; calcipotriol: 75.4 ± 8.1%, *p* = 0.040; alfacalcidol: 70.3 ± 5.4%, *p* = 0.006). The reason why paricalcitol showed no effect on *BACE1* gene expression remains speculative. Paricalcitol, a third generation analogue of vitamin D_2_, is an activator of VDR and is used for secondary hyperparathyroidism. When compared to calcitriol, different effects were observed e.g., a reduced stimulation of the intestinal calcium transport proteins or a reduced effect on calbindin expression [52]. Similar mechanisms might be responsible for an absent effect on *BACE1* expression in the presence of paricalcitol. However, the underlying mechanisms remain to be elucidated. 

On average, all of the analogues revealed a significant reduction in *BACE1* gene expression to 82.1% (mean analogues: 82.1 ± 4.6%, *p* = 0.005) (Figure 2E). In accordance with these results, analyzing BACE1 protein level by WB analysis, all vitamin D analogues revealed significant results. In the presence of calcifediol or the vitamin D_3_ and vitamin D_2_ analogues, BACE1 protein level was significantly reduced (Figure 2F and supplement Figure S3). 25(OH) vitamin D_3_, calcifediol, showed the most pronounced reduction in BACE1 protein level to 54.1% (calcifediol: 54.1 ± 5.7%, *p* = 0.004) (Figure 2F). The vitamin D_3_ analogues maxacalcitol, calcipotriol, and alfacalcidol revealed a 20 to 30% reduction in BACE1 protein level. For alfacalcidol, the decrease did not reach a significant level (maxacalcitol: 71.4 ± 5.8%, *p* = 0.020; calcipotriol: 68.9 ± 2.8%, *p* = 0.009; alfacalcidol: 77.6 ± 8.4%, *p* = 0.099). A significant reduction in BACE1 protein level to 63.2% and 74.3%, respectively, was also obtained for the vitamin D_2_ analogues paricalcitol and doxercalciferol (paricalcitol: 63.2 ± 4.2%, *p* = 0.005; doxercalciferol: 74.3 ± 5.4%, *p* = 0.025). All of the analogues averaged showed a significant decrease in BACE1 protein level to 71.1% (mean analogues: 71.1 ± 2.5%, *p* = 0.011) (Figure 2F), which is in line with the decreased *BACE1* expression to 82.1%.

### 2.4. Vitamin D Analogues Decrease γ-Secretase Processing of APP

**Figure 3:**
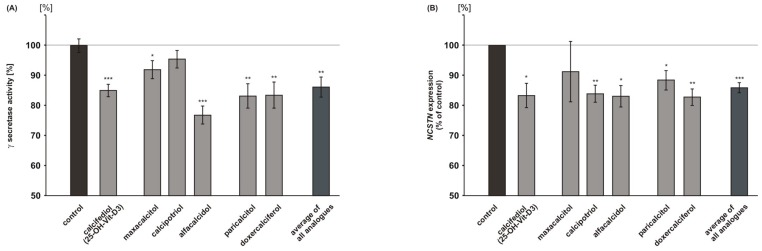
Effect of vitamin D and analogues on γ-secretase. (**A**) Analysis of γ-secretase activity in living SH-SY5Y wt cells (*n* ≥ 5). (**B**) mRNA level of the γ-secretase component nicastrin determined by RT-PCR analysis (*n* = 3). Error bars represent the standard error of the mean. Asteriks show the statistical significance calculated by unpaired Student’s *t* test (* *p* ≤ 0.05; ** *p* ≤ 0.01; *** *p* ≤ 0.001).

Beside the observed potency of vitamin D_3_ and vitamin D_2_ analogues to reduce amyloidogenic β-secretase dependent APP processing we analyzed whether γ-secretase activity, releasing Aβ peptides, is also affected in presence of these substances. As the effect for the vitamin D analogues was more prominent in cultured cells when compared to isolated membranes, and as effects on gene expression and protein level were found in case of β-secretase cleavage, we analyzed γ-secretase activity in metabolically active cells. When compared to cells treated with the solvent control calcifediol, maxacalcitol, alfacalcidol, paricalcitol, and doxercalciferol showed a significant reduction in γ-secretase activity, whereas calcipotriol did not affect γ-secretase activity (calcifediol: 85.3 ± 2.5%, *p* ≤ 0.001; maxacalcitol: 91.3 ± 3.0%, *p* = 0.039; alfacalcidol: 74.7 ± 3.3%, *p* ≤ 0.001; paricalcitol: 80.6 ± 4.0%, *p* = 0.0014; doxercalciferol: 80.2 ± 4.3%, *p* = 0.0017) (Figure 3A). All of the vitamin D analogues averaged significantly reduced γ-secretase activity to 86.1% (mean analogues: 86.1 ± 3.4%, *p* = 0.003). A significant reduction in gene expression of the γ-secretase component nicastrin was found for calcifediol, calcipotriol, alfacalcidol, paricalcitol, and doxercalciferol (calcifediol: 83.3 ± 4.1%, *p* = 0.015; calcipotriol: 83.9 ± 2.8%, *p* = 0.005; alfacalcidol: 83.1 ± 3.6%, *p* = 0.009; paricalcitol: 88.5 ± 3.2%, *p* = 0.023; doxercalciferol: 82.8 ± 2.7%, *p* = 0.003) (Figure 3B). Maxacalcitol showed a non-significant reduction in nicastrin mRNA levels to 91.3%. The average of all the analogues also revealed a significant reduction to 85.9% (mean analogues: 85.9 ± 1.7%, *p* ≤ 0.001), which is comparable to the observed reduction in secretase activity to 86.1%.

### 2.5. Vitamin D_3_ and Vitamin D_2_ Analogues Increase Aβ-Degradation

**Figure 4:**
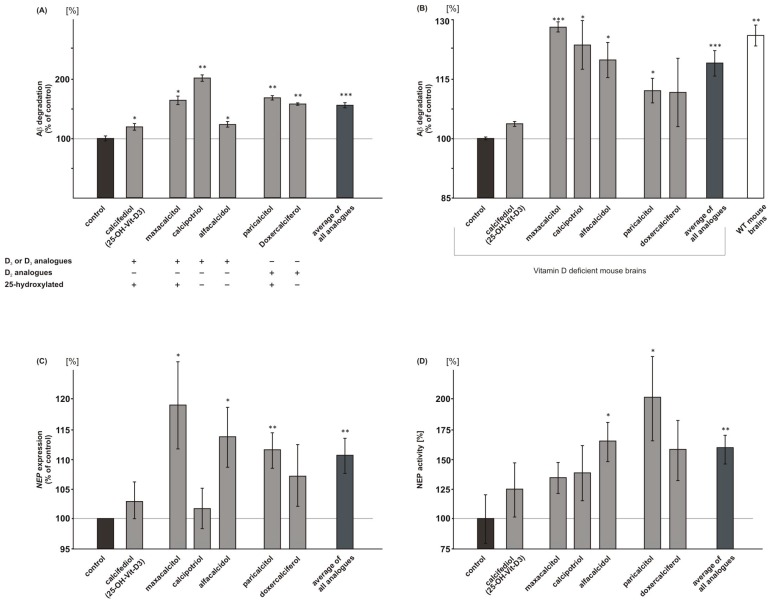
Effect of vitamin D and analogues on Aβ catabolism. Vitamin D and its analogues influence Aβ-degradation. Total Aβ-degradation in (**A**) mouse neuroblastoma N2a wt cells (*n* = 3) and (**B**) vitamin D deficient mouse brains (*n* = 5). Calcifediol and its analogues increased the Aβ-degradation compared to solvent control. (**C**) RT-PCR analysis of *NEP* expression in SH-SY5Y wt cells (*n* = 3). (**D**) NEP activity in N2a cells (*n* = 8). Error bars represent the standard error of the mean. Asteriks show the statistical significance calculated by unpaired Student’s *t* test (* *p* ≤ 0.05; ** *p* ≤ 0.01; *** *p* ≤ 0.001).

In order to investigate whether vitamin D_3_ and vitamin D_2_ analogues affect Aβ-degradation, we used mouse neuroblastoma N2a cells and treated these cells with vitamin analogues in presence of human synthetic Aβ peptides. Remaining synthetic Aβ peptides were detected by WB analysis, using an antibody recognizing human but not endogenous mouse Aβ. In the presence of 25(OH) vitamin D_3_, calcifediol, Aβ-degradation was significantly increased to 119.6% (calcifediol: 119.6 ± 4.5%, *p* = 0.034) (Figure 4A). The 1,25-hydroxylated and 1,24-hydroxylated vitamin D_3_ analogues maxacalcitol and calcipotriol increased Aβ-degradation to 164.5% and 202.1%, whereas the 1-hydroxylated vitamin D_3_ analogue alfacalcidol revealed an increase in Aβ-degradation that was similar to calcifediol (maxacalcitol: 164.5 ± 6.3%, *p* = 0.009; calcipotriol 202.1 ± 4.2%, *p* = 0.002; alfacalcidol: 123.5 ± 3.5%, *p* = 0.046) (Figure 4A). An increased Aβ-degradation was also found for vitamin D_2_ analogues. 1,25-hydroxylated paricalcitol elevated Aβ-degradation to 168.7%, 1-hydroxylated doxercalciferol to 158.2% (paricalcitol: 168.7 ± 2.6%, *p* = 0.002; doxercalciferol: 158.2 ± 2.8%, *p* = 0.003). The average of all vitamin D analogues showed a significant increase in Aβ-degradation to 156.1% (mean analogues: 156.1 ± 3.2%, *p* ≤ 0.001) (Figure 4A). We analyzed whether vitamin D_3_ and vitamin D_2_ analogues also have the potential to increase Aβ-degradation in vitamin D deficient mouse brains. Therefore, we incubated homogenates of vitamin D deficient mouse brains with vitamin D analogues in an ex vivo experiment. Maxacalcitol, calcipotriol, alfacalcidol and paricalcitol also significantly increased Aβ-degradation in case of vitamin D deficiency (maxacalcitol: 128.3 ± 1.3%, *p* = 0.001; calcipotriol: 123.8 ± 6.2%, *p* = 0.041; alfacalcidol: 120.0 ± 4.5%, *p* = 0.027; paricalcitol: 112.2 ± 3.1%, *p* = 0.043), whereas calcifediol and doxercalciferol did not affect Aβ-degradation in mouse brain homogenates of vitamin D deficient mice (Figure 4B). Again, on average, all of the analogues revealed a significant increase in Aβ-degradation in vitamin D deficient mouse brains to 119.2% (mean analogues: 119.2 ± 3.2%, *p* ≤ 0.001).

In line, RT-PCR analysis of treated human neuroblastoma cells SH-SY5Y revealed that vitamin D and all of the analogues tended to increase *NEP* expression, one of the main enzymes known to be involved in Aβ-degradation [13]. A significant increase in *NEP* gene expression was found for alfacalcidol, maxacalcitol, and paricalcitol (alfacalcidol: 113.8 ± 5.0%, *p* = 0.021; maxacalcitol: 119.1 ± 7.3%, *p* = 0.026; paricalcitol 111.6 ± 3.0%, *p* = 0.003) (Figure 4C). The average of all the analogues also showed a significant increase in *NEP* mRNA level to 110.7% (mean analogues: 110.7 ± 2.9%, *p* = 0.007). Similarly, vitamin D and the analogues tended to increase NEP activity (Figure 4D), which is again in line with elevated Aβ-degradation in presence of vitamin D or its analogues. Alfacalcidol and paricalcitol, which already showed a significant increase in *NEP* gene expression, also revealed a significant elevation in NEP activity (alfacalcidol: 165.2 ± 16.5%, *p* = 0.029; paricalcitol: 201.8 ± 35.3%, *p* = 0.029 (Figure 4D). All of the analogues averaged revealed a significant increase in NEP activity to 159.6% (mean analogues: 159.6 ± 12.0%, *p* = 0.0011). 

### 2.6. Influence of Vitamin D_3_ and Vitamin D_2_ Analogues on Inflammatory Processes

**Figure 5:**
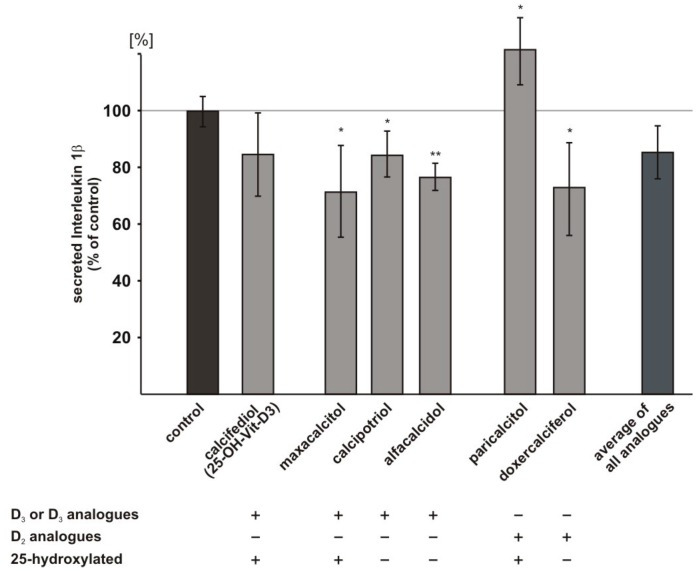
Effect of vitamin D and analogues on Interleukin-1β level. Interleukin-1β (IL-1β) level was determined by enzyme-linked immunosorbent assay (ELISA) technique (*n* ≥ 3). IL-1β was analyzed in SH-SY5Y wt cells incubated with 100 nM calcifediol or analogues compared to cells treated with the solvent control. Error bars represent the standard error of the mean. Asteriks show the statistical significance calculated by unpaired Student’s *t* test (* *p* ≤ 0.05; ** *p* ≤ 0.01).

As inflammation plays a crucial role in AD, we also investigated whether vitamin D analogues interfere with inflammatory processes. We selected cytokine Interleukin-1β (IL-1β), which is known to initiate inflammatory responses [53] and reported to be elevated in brains of AD patients [54] to analyze whether vitamin D_3_ and vitamin D_2_ analogues might reduce inflammatory responses and used ELISA technique to determine IL-1β level. The vitamin D_3_ analogues maxacalcitol, calcipotriol, and alfacalcidol significantly reduced IL-1β level to 71.4%, 84.4%, and 76.6%, respectively (maxacalcitol: 71.4 ± 9.3%, *p* = 0.044; calcipotriol: 84.4 ± 4.6%, *p* = 0.050; alfacalcidol: 76.6 ± 2.7%, *p* = 0.005) (Figure 5). Calcifediol and doxercalciferol also showed the tendency to decrease IL-1β level, however, the observed decrease did not reach statistical significance (calcifediol: 84.7 ± 8.4%, *p* = 0.167; doxercalciferol: 73.1 ± 9.4%, *p* = 0.054). Paricalcitol significantly increased IL-1β level (paricalcitol: 121.7 ± 6.8%, *p* = 0.044) (Figure 5).

## 3. Discussion

Besides the central role of vitamin D in calcium and phosphate homeostasis, vitamin D is discussed to have neuroprotective and anti-inflammatory properties [55,56] and is important for brain development influencing proliferation, differentiation, neurite outgrowth, and neuronal density [57,58]. Several studies also observed an association between cognitive impairment and vitamin D hypovitaminosis [59,60,61,62,63,64]. Moreover, Sutherland et al. reported a link between vitamin D and AD, describing a reduction in *VDR* mRNA levels in hippocampal CA1 and CA2 pyramidal cells of AD patients when compared to patients that were suffering from Huntington disease [65]. Studies during the past years suggest that lower vitamin D concentrations are associated with a substantially increased risk of all-cause dementia and AD [66,67,68]. Recently, Mokry et al. identified four single nucleotide polymorphisms (SNPs) in the vitamin D pathway that are significantly linked to AD [69]. In our previous study, analyzing mild vitamin D deficiency, we found increased Aβ peptide level caused by increased β-secretase cleavage of APP and decreased Aβ-degradation in vitamin D deficient mouse brains [29]. The aim of the present study was to investigate whether therapeutically used analogues of vitamin D_3_ and vitamin D_2_ also shows anti-amyloidogenic properties, and whether differences exist between single vitamin D analogues. In agreement with increased Aβ peptide level in the case of mild 25(OH) vitamin D_3_ deficiency, calcifediol, which is converted by 1α-hydroxylase CYP27B1 to active 1,25(OH)_2_ vitamin D_3_, calcitriol, decreased total Aβ level to 55.1% in human neuroblastoma cells. Also, the vitamin D_3_ analogues maxacalcitol, calcipotriol and alfacalcidol and the vitamin D_2_ analogues paricalcitol and doxercalciferol significantly reduced Aβ level. No significant differences were obtained between single vitamin D analogues. In line with our results, a similar reduction of approximately 50% in Aβ_40_ and Aβ_42_ peptides and a decrease in the number of amyloid plaques has also been shown in APP transgenic mice that were fed for five months with a vitamin D enriched diet [70]. Beside the potential of vitamin D to reduce Aβ level it has been recently shown that vitamin D_3_ protects against Aβ peptide cytotoxicity by reverting the Aβ_1–42_ induced reduction in the sphingosine-1-phosphate/ceramide ratio [26]. We found that vitamin D_3_ and analogues of vitamin D_3_ and D_2_ reduce secreted Aβ peptide level by decreasing amyloidogenic APP processing and by an elevation of Aβ-degradation. Impaired amyloidogenic APP processing in presence of vitamin D and vitamin D analogues is caused by a slight direct effect of these vitamins on β-secretase activity, and by a decrease in *BACE1* gene expression, accompanied by reduced BACE1 protein level. Besides transcriptional effects, it cannot be excluded that altered BACE1 protein stability in presence of vitamin D or vitamin D analogues might also contribute to the reduction in BACE1 protein level. BACE1 can be degraded by the lysosomal and ubiquitin-proteasome system (UPS) [71,72]. Interestingly, 1,25-dihydroxyvitamin D_3_ is involved in the regulation of numerous UPS genes, including ubiquitinating and deubiquitinating enzymes [73]. In this respect, is has to be mentioned that BACE1 degradation is dependent on ubiquitin carboxyl-terminal hydrolase L1, a deubiquitinating enzyme highly specific to neurons [74]. Furthermore, it could be recently shown that vitamin D deficiency down-regulates genes that are involved in protein catabolism [75]. However, so far it is unclear whether these mechanisms are also affected by vitamin D analogues, which has to be investigated in further studies. 

**Figure 6:**
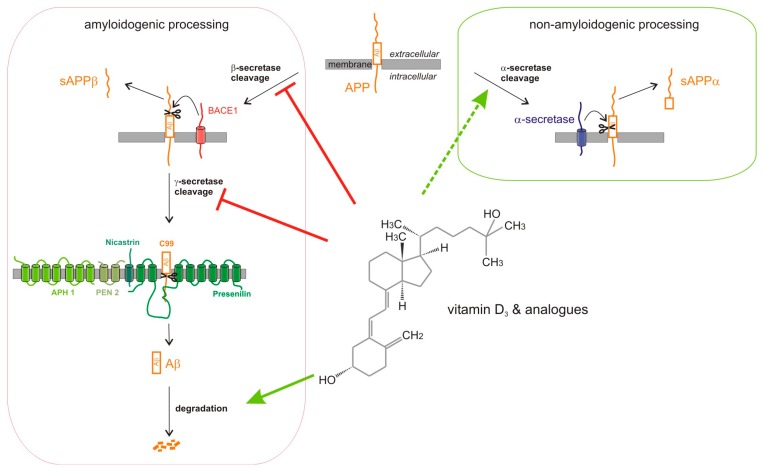
Model of the pleiotropic effects of vitamin D and analogues on Aβ-homeostasis. Vitamin D and analogues decrease amyloidogenic amyloid precursor protein (APP) processing by affecting β- and γ-secretase activity. The reduction of β-secretase activity is caused by a direct effect of vitamin D and its analogues on β-secretase activity combined with indirect effects on *BACE1* gene expression and total BACE1 protein level. The γ-secretase activity is reduced by decreased gene expression of nicastrin responsible for the maturation of the heterotetrameric γ-secretase complex. A stimulation of the non-amyloidogenic α-secretase processing of APP was found for 25(OH) vitamin D_3_ and the vitamin D_2_ analogue paricalcitol. Total Aβ level in presence of vitamin D and analogues are further reduced by increased Aβ-degradation.

In analogy to our observed effect on BACE1 protein level, Briones et al. found a 24% reduction in BACE1 protein level in old rats supplemented with vitamin D_3_ as compared to control animals [76]. In accordance to decreased BACE1 protein level in presence of vitamin D, silencing VDR in E16 primary rat cortical neurons increased mRNA and protein level of BACE1 [42], resulting in an increased intracellular Aβ_1–42_ level. The vitamin D induced reduction in β-secretase APP cleavage could be further substantiated ex vivo by supplementing vitamin D deficient and wt mouse brain homogenates with vitamin D_3_ or analogues of vitamin D_3_ and vitamin D_2_, indicating that both, patients with vitamin D hypovitaminosis and patients with a normal vitamin D status, might profit from vitamin D supplementation. Beside the effect of vitamin D and its analogues on β-secretase, we found significantly reduced γ-secretase activity in metabolically active cells in the presence of vitamin D or vitamin D analogues. In accordance to reduced γ-secretase activity, we found significantly reduced mRNA levels of nicastrin, indicating that altered gene expression of nicastrin, which is necessary for the maturation of the γ-secretase complex [77,78], contributes to the observed reduction in γ-secretase activity. Notably, the mRNA level of nicastrin have been shown to be increased in VDR silenced primary rat cortical neurons [42], thus supporting our results. In addition to the important role of β- and γ-secretase processing of APP in Aβ anabolism, Aβ level can be also decreased by elevated non-amyloidogenic α-secretase processing of APP. However, the effect of vitamin D and its analogues on α-secretase shedding is not as congruent as found for amyloidogenic β-secretase cleavage. In our study, calcifediol and paricalcitol were the only vitamin D species elevating non-amyloidogenic APP processing, whereas the other vitamin D analogues showed no significant effect. Gezen-Ak et al. even found an increase in mRNA and protein level of α-secretase ADAM10 in VDR siRNA-treated cortical neurons [42]. However, as additional metalloproteinases of the ADAM family have been identified as α-secretases [9,12,79], further studies in vitamin D treated, vitamin D deficient or VDR deficient cells are necessary to clarify the role of vitamin D and vitamin D analogues in α-secretase APP processing. Beside Aβ anabolism impaired Aβ-degradation is discussed to contribute to sporadic AD. Vitamin D_3_, as well as all analyzed vitamin D_3_ and vitamin D_2_ analogues, significantly elevated Aβ-degradation in mouse neuroblastoma cells. Increased Aβ-degradation in presence of vitamin D_3_ or its analogues was also obtained in vitamin D deficient mouse brains. Notably, we observed an increased gene expression of *NEP* and elevated NEP activity in the presence of vitamin D and its analogues, indicating that the Aβ-degrading enzyme NEP is affected by vitamin D and vitamin D_3_ and D_2_ analogues. These results are in line with the finding of Briones et al. reporting an increase in the protein level of NEP in vitamin D supplemented old rats [76]. Based on these findings, vitamin D and its analogues reduce total Aβ level by pleiotropic mechanisms affecting Aβ anabolism and Aβ catabolism (Figure 6). Each of the individual effects of the vitamin D analogues seem to be rather small and in some cases a significant level is not reached. However, all of the observed mechanisms result in a reduction of total Aβ level, which is highly significant and much more pronounced.

In summary, β-secretase cleavage is affected by a slight direct inhibitory effect on enzyme activity and a decrease in *BACE1* expression and BACE1 protein level. γ-secretase activity is reduced by decreased gene expression of the γ-secretase component nicastrin. Furthermore, vitamin D and its analogues increase Aβ-degradation, substantiating the important role of vitamin D and its analogues in Aβ homeostasis, and that supplementation with vitamin D_3_ or analogues of vitamin D_3_ and D_2_ might be protective against biological processes that are associated with AD. Notably, we found a correlation between β-secretase activity, the rate-limiting step in Aβ-generation, Aβ-degradation, and total Aβ level in presence of vitamin D or its analogues, as determined by Pearson correlation (r = 0.699, *p* = 0.081). In line, BACE1 protein level and Aβ-degradation correlates with the Aβ level (r = 0.746, *p* = 0.054). However, the Pearson correlation did not reach significance. Regarding vitamin D supplementation to prevent or treat AD, it is important to note that vitamin D_3_ also interferes with inflammatory processes that are known to contribute to AD. A significant increase in cytokine IL-1β level has been found in *post mortem* samples from AD patients with a maximum response in those brain regions, frontal cortex, and hippocampus, where AD neuropathology is most prominent [54]. Analyzing IL-1β, initiating inflammatory responses, we could show that vitamin D_3_ and vitamin D_2_ analogues, except paricalcitol, significantly reduced the pro-inflammatory IL-1β level. This finding is consistent with the recent findings by Raha et al. reporting that vitamin D_2_ attenuates Aβ_25–35_ induced pro-inflammatory cytokines, such as IL-1β, IL-6, and TNFα [27]. Furthermore, a recent study, describing the increased level of pro-inflammatory IL-1β and decreased level of anti-inflammatory IL-10 in old rats as compared to young animals, shows that this age-related change in inflammatory states was mitigated by vitamin D supplementation [76]. This effect seems to be not limited to pro-inflammatory cytokines, as it has recently been shown that 1,25-dihydroxyvitamin D_3_ upregulates IL-34 known to provide strong neuroprotective and survival signs in brain injury and neurodegeneration [80]. 

In conclusion, our results substantiate vitamin D supplementation as an approach to prevent or treat AD by reducing Aβ anabolism, elevating Aβ catabolism and the reduction of pro-inflammatory cytokines. The analyzed vitamin D analogues reduce secreted Aβ level with a similar potency, but differ partially in the effect strength of the underlying mechanisms, illustrating that individual AD patients might profit in a different extend of vitamin D analogues. AD patients with reduced anti-amyloidogenic β-secretase activity might benefit the most from supplementation with species like calcifediol and paricalcitol, as these vitamin D species were the only vitamins increasing non-amyloidogenic APP processing. According to our results, individuals with impaired Aβ-degradation might have the highest benefits from vitamin D analogues, like calcipotriol and maxacalcitol, showing the strongest effect on Aβ-degradation. In respect to β- and γ-secretase processing all of the vitamin D analogues revealed similar results, indicating that individuals with increased amyloidogenic secretase activities might benefit from the vitamin D analogues in a similar way. 

However, it has to be emphasized that the differences in the effect strength are rather small, and all of the analogues have been shown in respect to Aβ level to be similarly beneficial. Additionally, the use of vitamin D analogues in respect to AD seems to have no therapeutical advantage when compared to the use of calcifediol (or calcitriol), as no significant differences were found between vitamin D analogues and vitamin D. Therefore, further medical indications or pharmacological aspects of the vitamin D analogues should be taken into consideration as to which vitamin D analogue should be applied. Related to this, factors like plasma half-life of individual analogues, the ability to pass the blood-brain-barrier, the affinity for VDR as well as resorption, compatibleness, availability and potential side-effects have to be considered [43,44]. Especially pharmacological factors and the ability to pass the blood-brain-barrier might be even more relevant than the mechanistical differences of the individual analogues, which has to be further evaluated in in vivo experiments and clinical studies. 

## 4. Materials and Methods 

### 4.1. Chemicals and Reagents

Calcifediol, and its analogues, alfacalcidol, calcipotriol, doxercalciferol, maxacalcitol, and paricalcitol were purchased from MedChem Express (Monmouth Junction, NJ, USA), and all other chemicals used in this study were acquired from Sigma-Aldrich (Taufkirchen, Germany), if not stated otherwise.

### 4.2. Cell Culture and Mice

For the cell based experiments human neuroblastoma SH-SY5Y wt cells, SH-SY5Y APP695 cells and N2a cells were cultivated in Dulbecco’s Modified Eagle’s Medium (DMEM), containing 10% fetal calf serum (FCS, PAN-Biotech, Aidenbach, Germany) and 0.1% non-essential amino acid solution (MEM). For cultivating SH-SY5Y APP695, overexpressing the human APP695 isoform, 0.3 mg/mL hygromycin B (PAN-Biotech, Aidenbach, Germany) was added to the medium. The mouse neuroblastoma cell line N2a was maintained in DMEM/10% FCS/0.1% MEM supplemented with penicillin/streptomycin solution, 2 mM L-glutamine, and 1 mM sodium-pyruvat.

For the ex vivo experiments we used brains from female C57BL/6 wt mice (Charles River, Sulzfeld, Germany) and vitamin D deficient mice. All animal experiments were approved by the “Landesamt für Soziales, Gesundheit und Verbraucherschutz of the State of Saarland” (reference number 17/2011) following the national guidelines for animal treatment. To create the vitamin D deficit, C57BL/6 mice were fed as described, resulting in a 23% reduction in the 25(OH) vitamin D level [29]. After removing, the brains were washed in 0.9% sodium chloride and directly frozen in liquid nitrogen. To establish the homogenates, the mouse brains were slowly defrosted on ice, and afterwards were treated by Minilys (Peqlab, Erlangen, Germany) in HPLC-grade H_2_O. 

### 4.3. Vitamin D Incubations

#### 4.3.1. Cell Culture

To minimize the influence of 25(OH) vitamin D_3_ from serum, FCS in DMEM was reduced to 0.1%–2.5% 16 h before incubation, dependent on subsequent experiments. Incubation with 100 nM vitamin D or its analogues (dissolved in ethanol) was carried out for 24 h (8 + 16 h) in up to 2.5% FCS/DMEM. Controls were treated with ethanol in a final concentration of 1‰, corresponding to the concentration in the incubation media.

#### 4.3.2. Mouse Brains or Purified Membranes

Equal amounts of mouse brain homogenates or postnuclear fractions, adjusted using bicinchoninic acid assay, were incubated with 100 nM of vitamin D, its analogues or ethanol for 15 min at 4 °C. 

### 4.4. Determination of Protein Concentration

The determination of the protein concentration of the samples was performed by using the bicinchoninic acid assay (BCA), as described in [81].

### 4.5. Western Blot Experiments

Samples used for the WB experiments were adjusted to equal protein concentration in advance. 

For the determination of BACE1, cell lysates were prepared by lysing cells in 150 mM NaCl, 50 mM Tris/HCl pH 7.4, 2 mM EDTA, 0.1% NP-40, 0.1% Triton-X 100. For the determination of total secreted Aβ level and sAPPβ conditioned media were used. 

The following antibodies were used for WB analysis: W02 antibody (5 μg/mL; Millipore, Billerica, MA, USA), anti-sAPPβ: Mbs492139 (1:250; MyBioSource, San Diego, CA, USA), BACE1: ab2077 (1:1000; abcam, Cambridge, UK), anti-actin ab1801 (1:1000; abcam), anti-rabbit IgG HRP Conjugate W401B (1:5000; Promega, Mannheim, Germany) and anti-mouse P0260 (Dako, Hamburg, Germany). 

Aβ levels were detected by performing immunoprecipitation of conditioned media before WB analysis. Therefore, 20 μL protein G-Sepharose and W02 antibody (5 μg/mL) were used. 

Enhanced chemiluminescense (ECL)-method (Perkin Elmer, Rodgau-Jügesheim, Germany) was used to detect proteins. Densitometrically quantification was performed by using Image Gauge V3.45 software (Fujifilm, Düsseldorf, Germany).

### 4.6. Determination of Total Aβ-Degradation

For measuring the total degradation of Aβ, human synthetic Aβ_40_ (Bachem, Bubendorf, Switzerland) was added to murine samples. After a determined period of time, non-degraded human Aβ was detected by WB analysis using W02 antibody, which binds specifically to human Aβ, as described before [82]. Human Aβ differs in the W02 epitope compared to murine Aβ. Therefore, no endogenous produced Aβ is detected by the use of murine N2a cells. 

#### 4.6.1. Determination of Total Aβ-Degradation in N2a wt Cells. 

After cultivation of mouse neuroblastoma N2a wt cells in reduced FCS (0.1%) /DMEM for 6 h, cells were incubated with 100 nM vitamin D, its analogues or ethanol for 18 h and additionally 6 h in the presence of 0.5 μg/mL human synthetic Aβ_40_.

#### 4.6.2. Determination of Total Aβ-Degradation in Deficient Mouse Brains 

Deficient mouse brain homogenates were incubated as described earlier and treated for 1 h with synthetic Aβ_40_ in a final concentration of 1 μg/mL, 1 μM β-inhibitor, and 20 μM γ-inhibitor (Merck Millipore, Billerica, MA, USA).

### 4.7. Secretase Activity Assays

#### 4.7.1. Determination of α-, β- and γ-Secretase Activity in Living SH-SY5Y Cells 

Secretase activity assays were performed, as described before [82,83]. Following the incubation, cells were washed twice with prewarmed imaging solution containing 140 mM NaCl, 5 mM KCl, 8 mM CaCl_2_, 1 mM MgCl_2_, 20 mM HEPES, pH 7.4. Afterwards, 100 μL (α)/50 μL (β and γ) imaging solution mixed with 3 μM α-secretase substrate (Calbiochem, No. 565767), 20 μM β-secretase substrate (Calbiochem, No. 565758) or 6,25 μM γ-secretase substrate (Calbiochem, No. 565764) was added. The resulting fluorescence was determined continuously at excitation wavelengths of 340 ± 10 nm (α), 345 ± 5 nm (β), 355 ± 10 nm (γ) and emission wavelengths of 490 ± 10 nm (α), 500 ± 5 nm (β), 440 ± 10 nm (γ) under light preclusion and at 37 °C in a Safire2 Fluorometer (Tecan, Crailsheim, Germany).

#### 4.7.2. Determination of β-Secretase Activity in Isolated SH-SY5Y Membranes 

Measurements have been done as published in [23]. After homogenization of SH-SY5Y cells in sucrose buffer with Minilys (Peqlab, Erlangen, Germany) using ceramic beads, homogenates were adjusted to an equal protein amount by bicinchoninic acid assay and postnuclear fractions (PNFs) were isolated by sucrose density centrifugation. PNFs were incubated with calcifediol or its analogues for 15 min at 4 °C, and for pelleting membranes ultracentrifuged at 55,000 rpm for 75 min at 4 °C. Following, membranes were resuspended using glass beads in Minilys. For determination of β-secretase activity 20 μM specific β-secretase substrate (described above) was added to 125 μg of protein diluted 1:1 with 1 × PBS pH 4.5. Fluorescence was measured as described before.

### 4.8. RT-PCR Experiments

Quantitative real-time (RT) PCR has been done, as published in [19]. After isolation of total RNA from incubated SH-SY5Y cells using TRIzol Reagent (Thermo Fisher Scientific; Waltham, MA, USA), 2 μg RNA were reversed transcribed using the High-Capacity cDNA Reverse Transcription Kit (Thermo Fisher Scientific). RT-PCR using the Fast SYBR Green Master Mix (Applied Biosystems, Foster City, CA, USA) was performed on a PikoReal Real-Time PCR System (Thermo Fisher Scientific) and the following primers were used. β-actin: forward 5′-CTT CCT GGG CAT GGA GTC-3′, reverse 5′-AGC ACT GTG TTG GCG TAC AG-3′; TATA-box binding protein (TBP): forward 5′-CGG AGA GTT CTG GGA TTG T-3′, reverse 5′-GGT TCG TGG CTC TCT TAT C-3′; β-site APP-cleaving enzyme 1 (BACE1): forward 5′-GCC TAT GCT GAG ATT GCC AGG-3′, reverse 5′-GGA GAA GAG GTT GGG AAC GTG-3′; nicastrin: forward 5′-CTG TAC GGA ACC AGG TGG AG-3′, reverse 5′-GAG AGG CTG GGA CTG ATT TG-3′; neprilysin (NEP): forward 5′-GAT CAG CCT CTC GGT CCT TG-3′, reverse 5′-TGT TTT GGA TCA GTC GAG CAG-3′ (Eurofins MWG Operon, Eberberg, Germany). Two house-keeping genes (β-actin and TBP) were used to exclude RT efficiency differences. The obtained results were normalized to the β-actin/TBP ratio and the 2^−(ΔΔ*C*t)^ method was used to calculate expression changes.

### 4.9. Neprilysin Activity Assay

Measurement of NEP activity was performed as published in Miners et al. [84], with minor modifications utilizing the anti-NEP antibody AF1182 (R&D Systems, Minneapolis, MN, USA) and 5 μM MCA-RPPGFSAFK(DNP)-OH fluorogenic peptide substrate (R&D Systems, Minneapolis, MN, USA). Cells were chemical lysed in lysis buffer containing 0.5% TritonX-100, 20 nM Tris pH 7.4, 10% sucrose and fluorescence at an excitation wavelength of 320 ± 10 nm, and an emission wavelength of 405 ± 10 nm was measured in a Safire2 Fluorometer (Tecan, Crailsheim, Germany).

### 4.10. Enzyme-Linked Immunosorbent Assay (ELISA)

The level of cytokine IL-1β was measured in the medium of incubated SH-SY5Y wt cells using the Human IL-1 beta ELISA Kit (Abcam, Cambridge, UK).

### 4.11. Lactate Dehydrogenase (LDH) Activity Assay

The Cytotoxicity Detection Kit (LDH) from Roche (Basel, Schweiz), a colorimetric assay for the measurement of lactate dehydrogenase (LDH) release from cells, was used according manufacturer’s instructions to quantify cell death and lysis after incubation with different vitamin D concentrations.

### 4.12. Data Analysis

All quantified data represent an average of at least three independent experiments. Error bars represent standard error of the mean. Statistical significance was calculated using two-tailed Student’s *t* test, ANOVA, and post hoc Tests for multiple comparison analysis. The normality of the data distribution was tested with Shapiro Wilk test. Significance was set at * *p* ≤ 0.05; ** *p* ≤ 0.01 and *** *p* ≤ 0.001.

## Figures and Tables

**Table 1:** Comparison between β-secretase activity in supplemented (WT+; 100 nM calcifediol or its analogues) and unsupplemented (WT-) wildtype mouse brains and in supplemented (deficient+; 100 nM calcifediol or its analogues) and unsupplemented (deficient-) vitamin D deficient mouse brains.

Analogues	Statistical Test	WT+	WT+	WT+	WT-	WT-	Deficient+
		WT−	Deficient+	Deficient−	Deficient+	Deficient−	Deficient−
calcifediol	*t* test ^1^	0.028	0.000	0.000	0.005	0.000	0.026
	Bonferroni ^2^	0.039	0.000	0.000	0.099	0.001	0.450
alfacalcidol	*t* test	0.050	0.000	0.000	0.003	0.000	0.144
	Bonferroni	0.239	0.000	0.000	0.010	0.000	0.878
calcipotriol	*t* test	0.858	0.002	0.002	0.000	0.000	0.964
	Bonferroni	1.000	0.001	0.001	0.002	0.002	1.000
doxercalciferol	*t* test	0.573	0.499	0.014	0.145	0.000	0.040
	Bonferroni	1.000	1.000	0.038	0.873	0.005	0.226
maxacalcitol	*t* test	0.015	0.000	0.000	0.001	0.000	0.606
	Bonferroni	0.167	0.000	0.000	0.001	0.000	1.000
paricalcitol	*t* test	0.179	0.000	0.000	0.000	0.000	0.223
	Bonferroni	0.945	0.000	0.000	0.000	0.000	1.000

^1^ Student’s *t* test was used to compare two groups. ^2^ Bonferroni was used to compare more than two groups.

**Table 2:** Comparison of the effects of calcifediol and its analogues on β-secretase activity in either WT or vitamin D deficient mouse brains. No significant differences between calcifediol and the analogues tested with Post Hoc Bonferroni.

Analogues		WT	Deficient
calcifediol	alfacalcidol	1.000	1.000
	calcipotriol	0.466	0.967
	doxercalciferol	0.066	1.000
	maxacalcitol	1.000	1.000
	paricalcitol	1.000	1.000
alfacalcidol	calcipotriol	1.000	1.000
	doxercalciferol	0.515	1.000
	maxacalcitol	1.000	1.000
	paricalcitol	1.000	1.000
calcipotriol	doxercalciferol	1.000	0.158
	maxacalcitol	1.000	1.000
	paricalcitol	1.000	1.000
doxercalciferol	maxacalcitol	0.316	0.686
	paricalcitol	1.000	1.000
maxacalcitol	paricalcitol	1.000	1.000

## Supplementary Materials

Supplementary materials can be found at www.mdpi.com/1422-0067/18/12/2764/s1.
