# Supplementary figures and images for: Vitamin D and Its Analogues Decrease Amyloid-β (Aβ) Formation and Increase Aβ-Degradation
Source: Int J Mol Sci. 2017 Dec 19;18(12):2764. doi: 10.3390/ijms18122764 (PMC5751363; doi:10.3390/ijms18122764)

Figure S1:

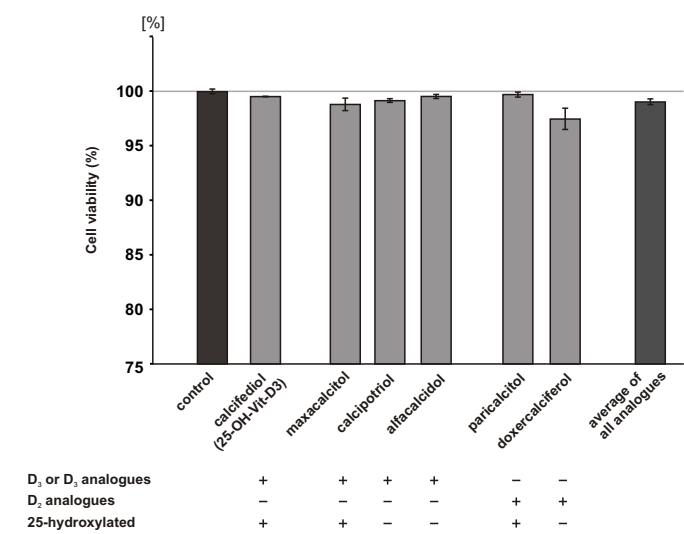

Figure S2:

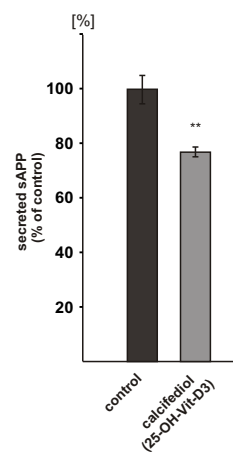

**Figure S3:**

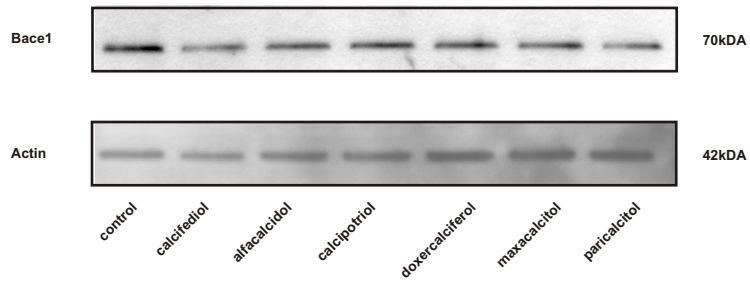

Supplement: Supplementary file 1 [file ijms-18-02764-s001.pdf]
